# Outcomes of Intra- versus Extra-Corporeal Ileocolic Anastomosis after Minimally Invasive Right Colectomy for Cancer: An Observational Study

**DOI:** 10.3390/jcm10020307

**Published:** 2021-01-15

**Authors:** Francesc Vallribera, Miquel Kraft, Meritxell Pera, Laura Vidal, Eloy Espín-Basany

**Affiliations:** Colorectal Surgery, Vall d’Hebron University Hospital, 08035 Barcelona, Spain; 20412fvv@comb.cat (F.V.); mkraft@vhebron.net (M.K.); mpera@vhebron.net (M.P.); l.vidal@vhebron.net (L.V.)

**Keywords:** colorectal cancer, minimally invasive right colectomy, intracorporeal anastomosis, extracorporeal anastomosis

## Abstract

Intracorporeal anastomoses (IA) are increasingly being used in colorectal surgery. Some data suggest that these might confer benefits compared with extracorporeal anastomoses (EA). The aim of this study is to compare the short-term complications associated with IA versus EA for minimally invasive right colectomy. This is a single-centre, retrospective study on a prospective database. Patients who underwent minimally invasive right colectomy for cancer between January 2017 and December 2019 were assessed for inclusion. The primary outcome was global 30-day morbidity. Overall, 189 patients were included, of whom 102 had IA. Global morbidity and medical complications were higher in patients with EA (23.5% vs. 40.2%, *p* = 0.014; 5.9% vs. 14.9%, *p* = 0.039, respectively). None of the patients with IA had non-infectious surgical wound complications, compared to 4.6% in the EA group (*p* = 0.029). No differences were found in anastomotic leakage (9.8% vs. 10.3%, *p* = 0.55). At multivariable analysis, EA was an independent risk factor for both surgical (OR = 3.71 95% CI: 1.06–12.91, *p* = 0.04) and overall complications (OR = 3.58 95% CI: 1.06–12.12, *p* = 0.04). IA lowers the risk for global, medical, and surgical complications with minimum risk for wound complications, without increasing the risk of AL.

## 1. Introduction

Colorectal cancer represents a very common condition, especially in developed regions [1,2]. Despite recent technical and technological advancements, anastomotic leak (AL) still represents one of the most feared complications in colorectal cancer surgery, reaching a 15% postoperative mortality [3], delaying subsequent oncological treatments and causing worse overall disease-free survival [4,5] as well as higher rates of local recurrence and cancer-related long-term mortality [6]. However, there is no universally accepted definition of AL [7,8], and surgeons show significant heterogeneity in what they define as AL [9]. AL rates vary between 1 and 30%, although in experienced colorectal units, it might be lower (3–6%) [10], depending on the definition, the tumour site, the surgical techniques used, or the individual surgeon performing surgery [11,12,13].

Several risk factors have been identified for AL, including age, preoperative nutritional status, or laparoscopic approach [14]. Stapled anastomosis has been reported to present higher rates of clinically relevant AL [14,15], but without increasing either mortality or length of stay (LOS).

Intracorporeal anastomosis (IA) might have some theoretical advantages over extracorporeal anastomosis (EA), such as less mesenteric traction, lower risk of twisted anastomosis, shorter skin incision for specimen extraction, and lower risk for developing incisional hernia [16]. Meta-analyses reported fewer infectious complications, lower rates of incisional hernia, and shorter LOS for IA [17,18], although no differences are seen for AL [19,20]. A recent randomized controlled trial [21] reported a quicker recovery of bowel function and lower postoperative pain for IA, without shorter LOS or differences in morbimortality and postoperative complications. The AL rate was higher in the IA group (8.6% vs. 2.9%) even if it did not reach statistical significance [21].

We herein report on a three-year experience at a high-volume colorectal unit, analysing the short-term clinical impact of IA vs. EA after minimally invasive right colectomy.

## 2. Materials and Methods

This is a single-centre, observational, retrospective study on a prospective database, which included patients operated on between January 2017 and December 2019 at Vall d’Hebron University Hospital (Barcelona, Spain). Approval from the ethical committee was obtained. This study was performed according to the Strengthening The Reporting of Observational Studies in Epidemiology (STROBE) Statement [22].

### 2.1. Eligibility Criteria

All consecutive patients undergoing minimally invasive right colectomy for cancer of the terminal ileum, appendix, or right colon were assessed for inclusion. Patients undergoing open surgery, those who needed conversion to open surgery, or patients with terminal stoma without an anastomosis were excluded. Patients with other than malignant diseases were excluded.

### 2.2. Surgical Technique

All procedures were performed or supervised by 6 surgeons at our institution. The decision on the suture type was made by the staff surgeon based on previous individual experience and clinical considerations. Three surgeons preferred EA and three preferred IA. Patients did not undergo mechanical bowel preparation and all of them received standard preoperative intravenous antibiotic prophylaxis.

The stapled anastomotic type was standardized, using side-to-side, either iso- or aniso-peristaltic ileocolic anastomosis, using a double reinforcement with two continuous absorbable and barbed (v-loc^TM^) sutures at the mesenteric and antimesenteric side. The distal ileum and transverse colon were divided with a 60 or 45 mm stapler device. The posterior running suture was performed first. Then, a 4 cm mechanically stapled ileocolic anastomosis was performed with a 60 or 45 mm stapler. Two different 60 mm stapler devices were used in the laparoscopic approach (Echelon^TM^, Endo Gia^TM^) depending on surgeon preference. A 45 mm stapler device was used in the robotic approach (EndoWrist^TM^). Haemostasis of the endoluminal suture line was revised, and the enterotomy was closed with a running barbed absorbable suture. Finally, an anterior suture with a running barbed absorbable suture was performed, covering the staple line. The mesentery was not approximated. The mini-laparotomy for specimen extraction was suprapubic (Pfannenstiel) in the IA group, whereas it was periumbilical or right transverse in the EA group, to allow the anastomosis to be performed. A wound protector was always used to extract the specimen or perform the EA (Alexis^®^, Rancho Santa Margarita, CA, USA).

### 2.3. Variables

The primary endpoint was 30-day global complication rate. Complications were graded according the Clavien–Dindo (CD) classification [23] and the Comprehensive Complication Index (CCI) score [24]. Secondary outcomes were: postoperative ileus, Surgical Site Infections (SSI), wound complications (wound infection, haematoma), Anastomotic Leak (AL), need for reoperation, evisceration, and medical complications (respiratory, cardiac, urinary). AL was graded with severity grade A–C [25]. Follow up was 30 days. All patients underwent an anastomotic leak detection program. This program consists of a detection of CRP (C-Reactive Protein) analysis on the third postoperative day, and repeated along with Procalcitonin level on the 4th day when CRP was higher than 140 mg/mL on the 3rd day. If at the 4th day, CRP was higher than 125 mg/mL or Procalcitonin levels were higher than 0.41 mg/mL, a CT scan was performed, irrespective of symptoms. All patients with clinical or radiological suspicion of AL were classified as having AL.

### 2.4. Statistical Methods

Categorical data are presented as absolute numbers and percentages, whereas continuous variables are presented as median with range. The Chi-squared test was used for the comparison of qualitative variables and Student’s *t* test for the analysis of quantitative variables. *p* values < 0.05 were considered statistically significant. Univariate analysis was performed to study the possible association between the type of anastomosis and the postoperative morbidity. Multivariate analyses were performed to identify factors associated with both overall and surgical complications.

## 3. Results

**Figure 1:**
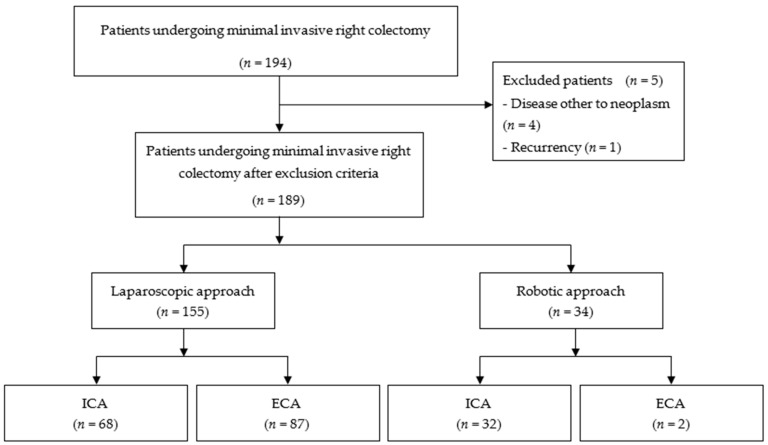
PRISMA flowchart. ICA: Intracorporeal anastomosis; ECA: extracorporeal anastomosis.

A total of 189 patients who underwent elective, minimally invasive right colectomy for neoplasm met the inclusion criteria, as shown in the flowchart (Figure 1). The median age was 75 years (Range: 39–96); 155 patients underwent laparoscopic and 34 robotic colectomies. IA was performed in 102 patients (54%), while EA in 87 (46%). No significant differences were found in the baseline characteristics with respect to the type of anastomosis, with the exception of EA being less commonly performed with robotic surgery (Table 1).

Global morbidity was higher in patients with EA, with a difference greater than 15% (23.5% vs. 40.2%, *p* = 0.014) and they had more medical complications (5.9% vs. 14.9%, *p* = 0.039) (Table 2). No significant differences were found for global surgical complications (17.8% vs. 27.6%, *p* = 0.1), SSI (2.9% vs. 6.9%, *p* = 0.31), AL (9.8% vs. 10.3%, *p* = 0.55), and need for reoperation (7.8% vs. 3.5%, *p* = 0.14). None of the patients in the IA group had a non-infectious surgical wound complication, compared to 4.6% in the EA group (*p* = 0.029). Patients with IA showed a trend toward a reduced incidence of postoperative ileus (10.8% vs. 20.7%, *p* = 0.06).

In the multivariable analysis, EA was an independent risk factor for both surgical complications (OR = 3.71, 95% CI: 1.06–12.91, *p* = 0.04) and overall morbidity (OR = 3.58, 95% CI 1.06–12.12, *p* = 0.04), whereas perioperative blood transfusions increased the risk of surgical complications (OR = 12.14, 95% CI 2.89–50.91, *p* = 0.001), and physiological POSSUM score increased the risk of overall morbidity (OR = 1.24, 95% CI 1.02–1.49, *p* = 0.033) (Table 3).

## 4. Discussion

Many studies have been published aimed at identifying the best anastomotic technique after right colectomy, usually focusing on AL, surgical complication rates, or need for reoperation. On this line, recent meta-analyses report different advantages for IA, such as shorter laparotomy incisions, shorter time to first flatus and bowel movement, shorter LOS, or a lower incisional hernia rate [17,18,19,20]. One meta-analysis, with more than 4000 patients included [18], reported lower rates of AL in IA, while others did not show any differences between IA and EA [17,19,20]. Our results are similar to these findings, without significant differences neither for global surgical complications, SSI, AL, nor the need for reoperation. Similar to the previously mentioned studies, we found no surgical wound complications in the IA group compared with four patients in the EA group.

The incision made for EA was performed as a peri-umbilical or right transverse laparotomy, whereas it was a Pfannenstiel incision in all IA patients. DeSouza et al. recommends a Pfannenstiel incision for laparoscopic colorectal surgery when possible [26]. Data from a recent meta-analysis [27] showed higher risk of incisional hernia rate in off-midline vs. midline incisions. Although we did not analyse incisional hernia rate because we only analysed short-term outcomes, three patients in the EA group presented evisceration which needed reoperation and two of them presented haematoma in the surgical wound. A long-term analysis would probably confirm a higher incisional hernia rate and could be a good target for future studies. Differences in incisional infection were not statistically significant. Of note, the SSI rates were lower than what is described in the literature for colorectal surgery, in both groups. This is the result of the VINCat Program for SSI prevention that has been established in Catalonia Region, and can also be attributed to the preoperative preparation used at our centre [28,29,30,31,32,33,34].

In our multivariate analysis, EA was an independent risk factor for surgical complications. These results are similar to the findings published by Shapiro et al. [35], in a comparative study of 191 patients, in which IA was associated with fewer overall postoperative complications, decreased rates of SSI, and fewer Clavien–Dindo III complications. In our multivariate analysis, the other risk factor for surgical complications was the need for blood transfusions. Neither drainage left at surgery, physiological POSSUM score, nor ASA score were risk factors for surgical complications.

An interesting aspect is the analysis of medical complications. Patients in the EA group presented higher global morbidity and more medical complications. EA remained an independent risk factor for overall complications in our multivariate analysis, as well as a higher POSSUM score. If we analyse the medical complications in detail, most of them are related to postoperative anaemia (9.2% vs. 1% in EA and IA, respectively), which could be related to higher blood loss during surgery. It might be hypothesized that the greater mesenteric traction to perform an EA might contribute to a greater risk of bleeding. Our results could be explained by the results published by Wu et al. [20], which described reduced blood loss in IA. This could be an interesting variable to consider in further studies, provided that it is adequately assessed and recorded.

Although the results in our study favour IA, it is important to underline that IA can be challenging even in expert hands [36]. A randomized controlled trial published by Allaix et al. [21] described higher AL rates in the IA group (8.6% vs. 2.9%), calling for more trials to investigate this further. A robotic approach might facilitate an IA technique in colorectal surgery [37,38]. From a technical perspective, robotic-assisted surgery facilitates suture techniques in all the surgical specialties in which it is currently used. Not surprisingly, in our study, the proportion of IA of the robotic approach group is higher than the proportion of IA of the laparoscopic approach group.

The present study has some limitations. It is a retrospective study, in which only short-term outcomes were assessed. A robotic approach, which was performed in more patients in the IA group, could be responsible for less surgical complications, although more studies are needed to corroborate it. Nonetheless, our study has a highly standardised operative technique, performed in a homogeneous group of colorectal cancer patients, making results more reliable.

## 5. Conclusions

This study showed a higher global morbidity in patients undergoing EA, with more medical and non-infectious surgical wound complications. No other differences were observed. Although an IA might be technically difficult, colorectal surgeons should consider this anastomotic technique in view of the associated benefits in the short-term. 

## Figures and Tables

**Table 1:** Baseline characteristics.

		Type of Anastomosis		
	All (189)	IA (102)	EA (87)	*p*
Gender, male, *n* (%)	93 (49.2)	51 (50)	42 (48,3)	0.66
Age, years, median (range)	77 (37–93)	77 (37–92)	76 (49–93)	0.7
ASA classification, *n* (%)				0.65
I	10	6	4	
II	103	57	46	
III	72	38	34	
IV	4	1	3	
POSSUM physiological score, median (range)	18 (11–32)	16 (12–30)	18 (11–32)	0.81
Surgical approach, *n* (%)				<0.001
Laparoscopy	155	68 (43.9)	87 (56.1)	
Robotic surgery	34	32 (94.1)	2 (5.9)	

ASA: American Society of Anaesthesiologists’ score; IA: intracorporeal anastomosis; EA: extracorporeal anastomosis.

**Table 2:** Morbimortality according to anastomotic type.

		Type of Anastomosis		
	All (*n* = 189)	IA (*n* = 102)	EA (*n* = 87)	*p*
Global morbidity, *n* (%)	59 (31.2)	24 (23.5)	35 (40.2)	0.014
Mortality, *n* (%)	6 (3.2)	4 (3.9)	2 (2.3)	0.53
Medical complications, *n* (%)	19 (10)	6 (5.9)	13 (14.9)	0.039
Cardiac complications	6 (3.2)	3 (2.9)	3 (3.5)	
Anaemia	9 (4.8)	1 (1)	8 (9.2)	
Respiratory complications	4 (2.1)	2 (2)	2 (2.3)	
Global surgical complications, *n* (%)	42 (22.2)	18 (17.8)	24 (27.6)	0.1
Surgical Site Infection	9 (4.8)	3 (2.9)	6 (6.9)	0.31
Anastomotic leakage, *n* (%)	19 (10)	10 (9.8)	9 (10.3)	0.55
Non-infectious surgical wound complications, *n* (%)	4 (2.1)	0	4 (4.6)	0.029
Evisceration	3 (1.6)	0	3 (3.4)	
Haematoma	2 (1)	0	2 (2.2)	
Postoperative ileus, *n* (%)	29 (15.3)	11 (10.8)	18 (20.7)	0.06
Postoperative ileus, *n* (%)	29 (15.3)	11 (10.8)	18 (20.7)	0.06
Need for reoperation, *n* (%)	11 (5.8)	8 (7.8)	3 (3.5)	0.14

IA: intracorporeal anastomosis; EA: extracorporeal anastomosis.

**Table 3:** Multivariable analysis for surgical and overall complications. Odds Ratio > 1 is associated with increased risk.

	Surgical Complications		Overall Complications	
Variable	OR (95% CI)	*p*	OR (95% CI)	*p*
POSSUM physiological score	1.08 (0.93–1.25)	0.315	1.24 (1.02–1.49)	0.033
Drain placement at surgery		0.666		0.679
No	Ref.		Ref.	
Yes	1.29 (0.4–4.15)		1.44 (0.26–7.97)	
ASA		0.219		0.121
ASA I	Ref.		Ref.	
ASA II	0.29 (0.06–1.44)		0.32 (0.03–3.51)	
ASA III	0.56 (0.12–2.65)		1.27 (0.13–12.2)	
ASA IV	3.1 (0.18–52.6)		3.19 (0.14–70.78)	
Blood transfusion		0.001		0.083
No	Ref.		Ref.	
Yes	12.14 (2.89–50.91)		4.02 (0.83–19.38)	
Anastomosis: technique		0.187		0.201
Hand-sewn	Ref.		Ref.	
Stapled	1.87 (0.74–4.75)		2.16 (0.66–7.04)	
Anastomosis: type		0.040		0.040
Intra-corporeal	Ref.		Ref.	
Extra-corporeal	3.71 (1.06–12.91)		3.58 (1.06–12.12)	

ASA: American Society of Anaesthesiologists’ score; OR: Odds Ratio; CI: Confidence Intervals. Statistically significant values in bold text.
